# Foreign Body in the Oral Cavity Mimicking a Benign Connective Tissue Tumor

**DOI:** 10.1155/2013/369510

**Published:** 2013-03-24

**Authors:** Divya Puliyel, Amir Balouch, Saravanan Ram, Parish P. Sedghizadeh

**Affiliations:** Ostrow School of Dentistry, University of Southern California, Los Angeles, CA 90089, USA

## Abstract

Foreign bodies may be embedded in the oral cavity either by traumatic injury or iatrogenically. The commonly encountered iatrogenic foreign bodies are restorative materials like amalgam, obturation materials, broken instruments, needles, and impression materials. This paper describes an asymptomatic presentation of a foreign body in the oral mucosa which clinically appeared like a benign connective tissue tumor.

## 1. Introduction

Foreign bodies implanted in the region of the oral cavity are described periodically in the dental literature, but recent reports are rare. This could be due to the more common use of rubber dams and techniques that avoid trauma and implantation in tissues. Often the foreign bodies reported are dental materials, metallic projectiles, and glass [1]. In most documented instances, patients present with oral pain and signs of inflammation with purulent discharge [2]. Reports of asymptomatic foreign bodies affecting the oral cavity are rarely reported in the literature. This paper describes a patient with an asymptomatic lesion that presented as a benign tumor-like mass, but which was later found to be rubber-based impression material implanted in the mucobuccal fold.

## 2. Case Report

**Figure 1:**
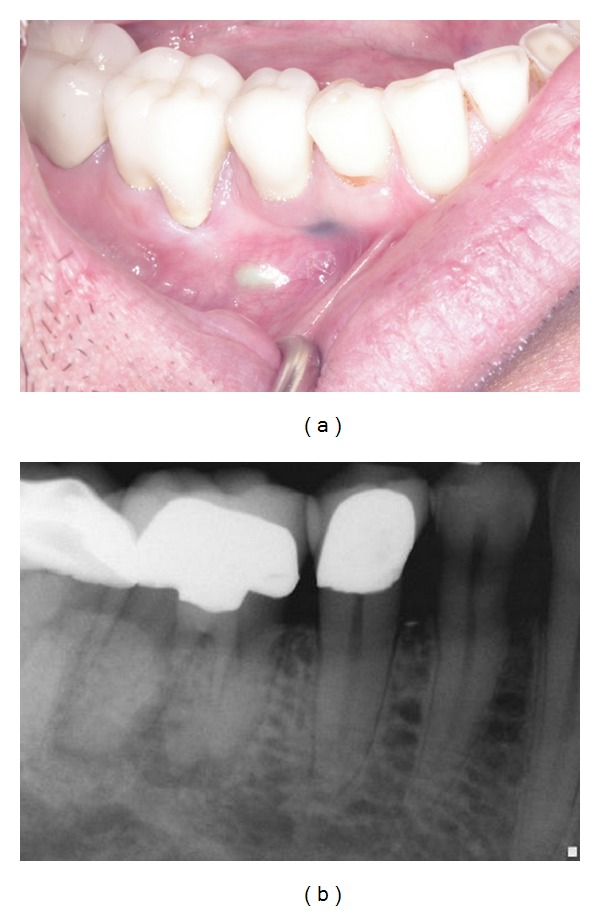
(a) 5 mm by 3 mm yellowish-white mass in the mucobuccal fold region of the lower right second premolar. A bluish-purple hyperpigmentation similar to the appearance of an amalgam tattoo is also noted on the alveolar process between the premolars. (b) Radiograph shows no radiopacity in the region of the premolars. The lingual tori can be seen as well-defined areas of radiopacity overlying the tooth roots of the molars.

**Figure 2:**
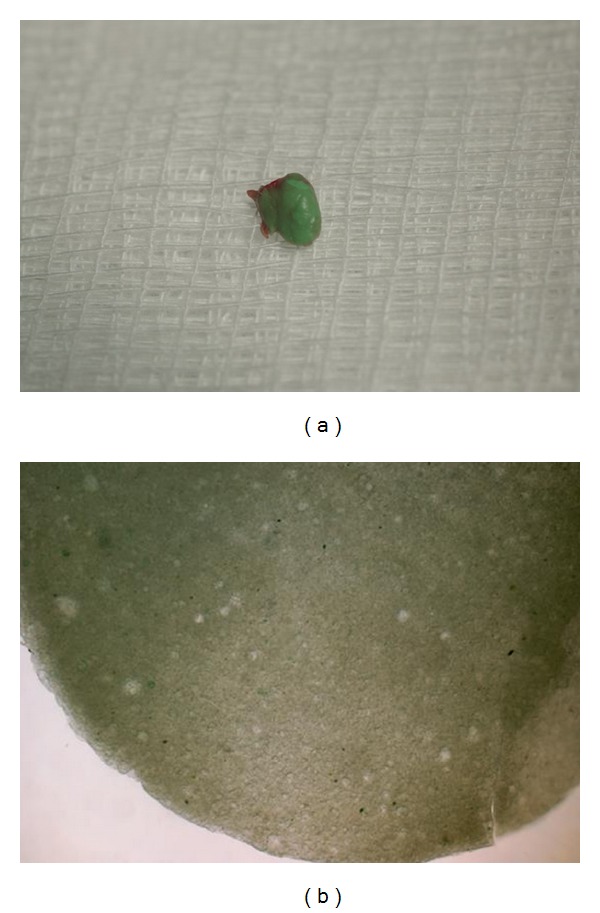
(a) 5 mm by 3 mm well-defined green mass with a thin membranous capsule. (b) Histopathology shows an acellular pale green amorphous material with multiple areas of vacuole formation.

A 51-year-old man presented to the Ostrow School of Dentistry, University of Southern California, with the chief complaint of a recently dislodged porcelain-fused-to-metal (PFM) crown affecting the right maxillary first molar. This PFM had been placed five years priorly. The patient's past dental history included multiple restorative procedures. Medical and social history was noncontributory. On routine clinical examination of his oral cavity, a well-defined submucosal mass was identified in the region of the lower right first and second premolars. On stretching the lip and cheek, a 5 mm by 3 mm yellowish-white nodule was visible in the mucobuccal fold region of the lower right second premolar (Figure 1(a)). The mass was soft to rubbery-firm on palpation, with no surrounding induration. It was minimally compressible and margins were well defined. The nodule was mobile and could be easily moved with the back of a mouth mirror. The patient experienced no pain on palpation and was unaware of the presence or duration of the lesion. The patient was referred to the Oral Medicine Clinic for lesion evaluation and definitive diagnosis. An intraoral periapical radiograph was taken of the region (Figure 1(b)). Vitality testing of the teeth in the region was inconclusive. Based on clinical and radiographic findings, the differential diagnosis for the mass included lipoma, granulation tissue, scarred parulis, fibroma, leiomyoma, neuroma, schwannoma, and foreign body granuloma. Lipoma was considered most likely because of the yellow color, morphology, and consistency on clinical palpation and absence of symptoms. The patient consented to biopsy for definitive diagnosis of the mass and removal. Excisional biopsy was performed under local anesthesia. The mass was bluntly dissected and removed* in toto *and placed in 10% formalin for routine histopathologic examination. On gross examination, the mass was greenish in color and there was evidence of a thin overlying fibrous capsule (Figure 2(a)). Microscopic examination revealed an acellular and amorphous mass with no signs of cellular tissue or associated inflammation (Figure 2(b)). The final diagnosis was intramucosal foreign body consistent with rubber-based impression material. Upon further questioning and discussion with the patient's previous dentist, it was confirmed that the patient had previous amalgam restorations removed in the region and subsequent crown lengthening and PFM placement. Impression material used for PFM fabrication was polyvinyl siloxane.

## 3. Discussion

All reported cases of impression material presenting as a foreign body describe an associated inflammatory reaction [3–6]. Dental impression materials are manufactured to be biocompatible and have minimal cytotoxic effects. Studies have shown that there is a low probability of allergic or toxic reactions [7]. Cytotoxic tests on cell cultures have shown that polyethers are more toxic than vinyl polysiloxanes [8]. The biocompatible nature of the material may explain the pseudocapsule formation around the excised lesion in our patient as the body's mechanism to wall off the foreign body. Radiographs aid in the location of foreign bodies if the material is dense enough to appear radiopaque. If the material is radiolucent, it may be identified if it is present in considerable thickness or density [2]. Foreign body implanting within the mucosa may be the result of material being forced through tissue traumatized during crown preparation. It is suggested that after taking impressions, all tissues which involve exposed bone or traumatized soft tissue must be well irrigated to remove any impression material residue [6]. Dental clinicians should be aware that intramucosal foreign body can be an incidental finding on intraoral examinations and can mimic the appearance of a benign and well-defined connective tissue tumor.

## References

[1] Arora BK, Ruprecht A (1978). Foreign body in tongue. *Oral Surgery Oral Medicine and Oral Pathology*.

[2] Price C, Whitehead FI (1972). Impression materials as foreign bodies. *British Dental Journal*.

[3] Eliasson ST, Holte NO (1979). Rubber-base impression material as a foreign body. Report of a case. *Oral Surgery Oral Medicine and Oral Pathology*.

[4] Kent WA, Shillingburg HT, Tow HD (1988). Impression material foreign body: report of a case. *Quintessence International*.

[5] Giusto TJ (2006). Localized severe periodontitis associated with retained impression material and root proximity: report of a case. *Journal of the New Jersey Dental Association*.

[6] Garey RC, Narang R (1976). An unusual foreign body in the buccal vestibule: report of a case. *Oral Surgery Oral Medicine and Oral Pathology*.

[7] Sydiskis RJ, Gerhardt DE (1993). Cytotoxicity of impression materials. *The Journal of Prosthetic Dentistry*.

[8] Roberta T, Federico M, Federica B, Antonietta CM, Sergio B, Ugo C (2003). Study of the potential cytotoxicity of dental impression materials. *Toxicology in Vitro*.

